# The ability of Psychosocial Care Centers specialized in Alcohol and Drugs to handle crises*

**DOI:** 10.1590/1518-8345.6167.3849

**Published:** 2023-06-02

**Authors:** Marianna Martins Pierini, Gabriella de Andrade Boska, Heloísa Garcia Claro, Priscilla de Oliveira Luz, Márcia Aparecida Ferreira de Oliveira

**Affiliations:** 1 Universidade Federal de São Paulo, Comissão de Residência Multiprofissional/COREMU, São Paulo, SP, Brasil.; 2 Universidade Federal do Rio Grande do Sul, Escola de Enfermagem, Porto Alegre, RS, Brasil.; 3 Universidade Estadual de Campinas, Faculdade de Enfermagem, Campinas, SP, Brasil.; 4 Universidade de São Paulo, Escola de Enfermagem, São Paulo, SP, Brasil.

**Keywords:** Crisis Intervention, Substance Abuse Treatment Centers, Outcome Assessment, Health Care, Homeless Persons, Substance-Related Disorders, Mental Health, Intervención en Crisis, Centros de Tratamiento de Abuso de Sustancias, Evaluación de Resultados en la Atención de Salud, Personas en Situación de Calle, Trastornos Relacionados con el Consumo de Sustancias, Salud Mental, Intervenção na Crise, Centros de Tratamento de Abuso de Substâncias, Avaliação de Resultados em Cuidados de Saúde, Pessoas em Situação de Rua, Transtornos Relacionados ao Uso de Substâncias, Saúde Mental

## Abstract

**Objective:**

to assess the ability of 24-hour Psychosocial Care Centers specialized in Alcohol and Other Drugs to handle the users’ crises in comprehensive care.

**Method:**

a quantitative, evaluative, and longitudinal study was conducted from February to November 2019. The initial sample consisted of 121 users, who were part of the comprehensibly care in crises by two 24-hour Psychosocial Care Centers specialized in Alcohol and other Drugs in downtown São Paulo. These users were re-evaluated 14 days after admission. The ability to handle the crisis was assessed using a validated indicator. The data were analyzed using descriptive statistics and regression of mixed-effects models.

**Results:**

67 users (54.9%) finished the follow-up period. During crises, nine users (13.4%; p=0.470) were referred to other services from the health network: seven due to clinical complications, one due to a suicide attempt, and another for psychiatric hospitalization. The ability to handle the crisis in the services was 86.6%, which was evaluated as positive.

**Conclusion:**

both of the services analyzed were able to handle crises in their territory, avoiding hospitalizations and enjoying network support when necessary, thus achieving the de-institutionalization objectives.

## Abstract (es)

**Objetivo:**

evaluar la capacidad de los Centros de Atención Psicosocial de Alcohol y Otras Drogas 24 horas para manejar situaciones de crisis de las personas que consumen AOD en la atención integral.

**Método:**

estudio cuantitativo, evaluativo y longitudinal, realizado de febrero a noviembre de 2019. La muestra inicial estuvo compuesta por 121 personas que consumen AOD, que recibieron atención integral en situaciones de crisis en dos Centros de Atención Psicosocial para Alcohol y Otras Drogas 24 horas en el centro de São Paulo. Los mismos fueron reevaluados después de 14 días de atención. La capacidad para manejar la crisis se evaluó mediante un indicador validado. Los datos se analizaron utilizando estadísticas descriptivas y modelos de regresión de efectos mixtos.

**Resultados:**

sesenta y siete personas que consumen AOD completaron el *follow-up* (54,9%). Durante la atención de las situaciones de crisis, nueve personas que consumen AOD (13,4%; p=0,470) fueron derivadas a otros servicios de la red de salud: siete por complicaciones clínicas, una por intento de suicidio y una por hospitalización psiquiátrica. La capacidad de los servicios para manejar situaciones de crisis fue del 86,6%, fue considerada positiva.

**Conclusión:**

los dos servicios evaluados fueron capaces de manejar situaciones de crisis en su área de influencia, evitando internaciones y contando con el apoyo de la red cuando fue necesario, logrando así los objetivos de desinstitucionalización.

## Abstract (pt)

**Objetivo:**

avaliar a capacidade dos Centros de Atenção Psicossocial Álcool e outras Drogas 24 horas em manejar situações de crise dos usuários no acolhimento integral.

**Método:**

estudo quantitativo, avaliativo e longitudinal, realizado de fevereiro a novembro de 2019. A amostra inicial foi composta por 121 usuários, acolhidos integralmente em situações de crise por dois Centros de Atenção Psicossocial Álcool e outras Drogas 24 horas do centro de São Paulo. Estes foram reavaliados após 14 dias de acolhimento. A capacidade de manejar a crise foi avaliada por um indicador validado. Os dados foram analisados por estatística descritiva e por regressão de modelos de efeitos mistos.

**Resultados:**

sessenta e sete usuários concluíram o follow-up (54,9%). Durante o acolhimento às situações de crise, nove usuários (13,4%; p=0,470) foram encaminhados para outros serviços da rede de saúde: sete por complicações clínicas, um por tentativa de suicídio e um para internação psiquiátrica. A capacidade de manejo das situações de crise pelos serviços foi de 86,6%, avaliada como positiva.

**Conclusão:**

os dois serviços avaliados foram capazes de manejar situações de crise no próprio território, evitando internações e tendo apoio da rede quando necessário, atingindo assim, os objetivos da desinstitucionalização.

**Highlights:**

**(1)** To our knowledge, this is the first study to evaluate CAPS AD’s ability to handle crises.

**(2)** The ability to handle crises of the CAPS AD from downtown São Paulo was 86.6%.

**(3)** Of the nine users referred to other services, only one evolved to hospitalization.

## Introduction

A mental health crisis can be defined as a critical moment characterized by disrupting a person’s organization in its psychological, social, biological, spiritual, or cultural dimensions^(1)^. A crisis can be triggered by various situations beyond a person’s limits. It usually involves a context signaled by changes, losses, and threats. Also, it is often marked by intense anxiety, uncertainty, unpredictability, and possible violence, due to the suffering expressed far beyond the psychiatric symptomatology^(2)^.

In Alcohol and Other Drugs (AOD) clinical management, crises can be relieved or worsened due to the effect of the substance(s) of choice or to their absence. Generally, characteristics such as craving, intoxication, overdose, and withdrawal symptoms are observed, in addition to the difficulty establishing interpersonal relationships, issues commonly related to the reduction of length of stay in the care spaces^(3)^.

Although not always qualified as urgent, crisis moments require immediate admission^(1,4)^. For more than a decade, the World Health Organization (WHO) has recommended that these services be preferentially carried out in the community context and by teams or specialized mental health services; however, these resources are still not sufficiently available or structured at the global level^(3-5)^.

A systematic literature review corroborated by recent national and international studies found that the lack of specialized and trained services for this management, the reproduction instances of coercive, authoritarian, and biomedical asylum practices, the failures in the articulation of the network, and the influences of the family and the community due to the need for hospitalization are some impasses for crisis care not to be successful in extra-hospital assistance^(2,4,6-7)^.

In the context of psychoactive substance use, it is common for users’ access to community mental health services to be relatively lower when compared to other mental disorders, due to the specific characteristics of their needs^(3)^. In addition, a longitudinal analysis carried out in the United States identified that the proportion of alternative community services for managing crisis situations in this population group was reduced by almost 10% in the country in seven years^(8)^.

Even in scenarios such as Italy, the Netherlands, and Brazil, which implemented similar deinstitutionalization models with a focus on community care, the assistance provided for crises is still one of the most critical points in mental health care, which evidences the need to evaluate this practice^(2,4)^.

In Brazil, crises care was reorganized after the Brazilian Psychiatric Reform with the 24-hour Psychosocial Care Centers (*Centros de Atenção Psicossocial*, CAPS III) in their different care modalities for adults with general mental health problems (CAPS III) and users of alcohol and other drugs (CAPS AD III). These services assume in-hospital extra-care in mental health, with the handling of crises among them. For this purpose, their structure has beds for comprehensive admission (day and night shifts), varying from six to 12 days depending on the service territory characteristics. They allow the inpatients to stay up to 14 days, with possible time extensions, considering the needs inherent to each case^(9)^.

Studies that characterized comprehensive care at CAPS AD III in different Brazilian regions identified that the bed occupancy rate varies from 86% to 100% and that, after discharge, from 22.6% to 36.3% of the users return in new crises, emphasizing recurrence and complexity in the management of these cases, especially in contexts of social vulnerability^(2,10)^.

The Ministry of Health (*Ministério da Saúde*, MS) considers that “successful admissions at crisis moments are essential to comply with the CAPS objectives, namely: treat severe and persistent mental disorders and avoid hospitalization”^(11)^. In this sense, the CAPS’ ability to handle crises is considered one of the main indicators to guarantee the space of these services in the Psychosocial Care Network (*Rede de Atenção Psicossocial*, RAPS), especially in this historical political moment marked by setbacks and devaluation of Brazilian psychosocial care^(12)^.

Indicators are important information tools in mental health; however, it is unusual to find standardized programs in the scientific literature because they are generally associated with specific public systems for different realities, which weakens the evaluation process, especially in low-income countries^(13)^.

Internationally, the assessments of care services for drug users are based on programs and policies specific to each country^(3)^. Some common indicators ensure crisis care exclusively in the community environment and no relapses (new crises)^(4,14)^. Similarly, in Brazil, 24-hour CAPS evaluation indicators have been validated recently. The ability to handle crises was included among them. This indicator measures the number of users in crisis referred by the services during the month, divided by the total number of users in crisis admitted beds, as well as the CAPS III’s ability to handle crises^(15)^.

Adapting this indicator to the CAPS AD III, this evaluation can generate diverse evidence about psychosocial care so that managers, health professionals, and users can appropriate these data and thus guarantee adequate investments and guidelines that subsidize extra-hospital care for AOD users.

To bridge the gap in the assessment of crisis management by community services, devoted to caring for psychoactive substance users, this study proposes to evaluate the CAPS AD III’s ability to handle crisis situations for users in comprehensive care.

## Method

### Research design

The current study has an evaluative and longitudinal design and a quantitative approach. It sought to evaluate the “CAPS AD III’s ability to handle crises” as a result indicator and to verify the changes and effects obtained from comprehensive care at the following moments: T_0_ - full admission; and T_1_ - after 14 days (time established by ministerial ordinance for permanence in full admission). Evaluation indicators aim at supporting objective changes in the system, analyzing the presence or absence of the effect after the observation.

The Strengthening the Reporting of Observational Studies in Epidemiology (STROBE) checklist for observational studies was used as a guide to writing this article^(16)^.

### Setting

This study was conducted in two CAPS AD III from the central region of the municipality of São Paulo - SP, Brazil.

Both services treat similar population groups comprised of people with problematic AOD use and living in extreme social vulnerability. The services have from eight to nine beds for comprehensive care, with an occupancy rate between 80.6% and 100% (data provided by the service). Care is offered by a multi professional team, with 24-hour permanence of the Nursing team.

These services were chosen as the research scenario because they are a reference for a territory of significant complexity regarding social and health needs, which concentrates almost 50% of the street population in São Paulo. In addition to that, it is in this region that one of the largest public scenarios of psychoactive substance use is found, known as “use setting in the Light region” or “crackland”^(17)^.

### Sample

Sample calculation was based on longitudinal studies with the same analysis object (AOD users undergoing treatment) and resorted to measures similar to those of the current study (days of substance use). One of the closest analyses significantly improved the users, with 0.06 points and a standard deviation of 0.165 according to the pre-and post-follow-up difference of means^(18)^. Therefore, by considering the same or a larger difference and adopting 95% analysis power, the sample calculation obtained corresponded to 101 participants at the end of the follow-up period.

Considering a 50% probability of sample losses, it was initially foreseen to include 152 subjects; however, the inclusion capability and final initial sample of this study totaled 121 participants. These subjects were included using the convenience approach.

### Inclusion, exclusion, and discontinuity criteria

The following were adopted as inclusion criteria: minimum age of 18 years old, being in due clinical conditions to answer the interview according to the assessment of the CAPS AD III teams, especially to mental status changes (psychomotor agitation, hallucinations, etc.), and being under the influence of psychoactive substances or with withdrawal symptoms that precluded participation in the research.

The subjects excluded were those that failed to fully answer the data collection instrument using the “Complete Analysis” perspective^(19)^. The discontinuity criteria considered in the research corresponded to those individuals that did not answer the interview in the follow-up period (T_1_).

### Variables and data collection instrument

We used two digital forms for each research moment (T_0_ and T_1_) as data collection instruments, which were accessed via a tablet or cell phone by the data collection team (main researcher, a post-PhD researcher, and female scientific initiation students).

The researchers prepared the instrument with synchronous storage of the information in a safe server at the University of São Paulo. Access to the data was only possible by using an exclusive and periodically updated password, according to the research data protection standards.

The form for T_0_ consisted of identification and sociodemographic variables, as well as related to substance use in the last 30 days. In the form corresponding to T_1_, in addition to the repetition of variables subjected to change collected at T_0_, such as substance use, the researchers added questions about the period in comprehensive admission, as well as the following question to answer the result indicator: the CAPS AD III’s ability to handle the crisis: During the full admission period at the CAPS AD III, have you been referred to any other service such as a general hospital, a psychiatric hospital, and urgency and emergency services, among others? Please indicate the reason.

Cross-validation of this information was performed in the participants’ medical charts. Subsequently, the following indicator was calculated: Number of users in crisis referred by the services/Total number of users in crisis admitted to the beds.

### Data collection

Data collection was carried out from February to November 2019. The researchers collected data using in-person interviews with users that had been comprehensively admitted to the CAPS AD III selected. The data collection was performed by applying the research instrument comprised of two forms described in the previous topic.

The users admitted in crisis by the CAPS AD III were approached on the premises of the services after stabilization of their condition, on the same admission day or the next day, depending on the situation. Participation in the research was by invitation and volunteer. The participants were interviewed after agreeing to and signing the Free and Informed Consent Form (FICF), with the application of the data collection form corresponding to T_0_.

At the end of this stage, a new interview was scheduled for 14 days later, with its date and time recorded in the medical chart and on a specific sheet of paper handed into the participant. The researchers returned to the CAPS AD III for the follow-up and to apply the T_1_form.

At this time, we carried out an active search for participants for up to five attempts at personal or family contact or through interlocution by the CAPS AD III professionals within a maximum period of 4 days after the date scheduled for the T_1_ interview. Those users not found and/or who did not participate in the follow-up period met the criteria for being excluded from the research.

The interviews lasted a mean of 30 minutes, and secrecy was ensured for the participants.

### Data analysis

The data were analyzed in the R software, version 3.5.1. The numerical variables were presented as central tendency and dispersion measures [mean and standard deviation (SD)] and the categorical variables using absolute and relative frequencies.

The longitudinal analysis was performed to verify the individual change over time for each variable under study, considering the variation of repeated measures over time. Linear regression models of mixed effects were applied for numerical variables. A generalized linear model of mixed results for ordinal and binary variables was adjusted with the cumulative and ordinary logistic link functions. Firth correction was applied when the contingency tables for these variables had cells with zero cases. A 95% confidence interval and a different parameter of p-value < 0.05 were considered.

As an evaluative parameter for the ability to handle crises, we used a maximum rate of 20% of referrals; in other words, if the services carried out up to 20% of referrals of the users admitted, they can be considered capable of handling these situations. For this definition, we based ourselves on the scientific literature in the area^(2,7,10)^ and the parameters expected by public policies. We did not find specific studies that used this measure, which was not defined in the validation study^(15)^.

### Ethical aspects

This study was approved by the Research Ethics Committees of the Nursing School at the University of São Paulo (2,759,176/2018) and the São Paulo Municipal Health Department (2,832,670/2018). All the participants signed the FICF.

## Results

Of all 121 users included in the cohort (T_0_), 67 (T_1_) finished the follow-up period (55.4%). The profile of those admitted in crises at the CAPS AD III corresponded to men (81.8%) (two participants identified themselves as transgender), with a mean age of 44 years old (SD=10.3), living on the streets (75.3%), without work activity (66.9%), with a mean of eight years of study (SD=3.8) and monthly incomes below one minimum wage (62.9%), most of the times, coming from social benefits (60.7%). All the information is described in Table1.

**Table 1 -:** Characteristics of the users admitted at the Psychosocial Care Centers specialized in Alcohol and Other Drugs at baseline. São Paulo, SP, Brazil, 2019

Variables	n (121)	%
**Gender**		
Male	97	80.2
Female	24	19.8
**Sexual orientation**		
Heterosexual	106	87.6
Bisexual	12	9.9
Homosexual	2	1.7
Asexual	1	0.8
**Race/Skin color (self-declared)**		
Brown	58	47.9
White	32	26.4
Black	27	22.4
Indigenous	1	0.8
Other	3	2.5
**Marital status**		
Single	88	72.7
Divorced	16	13.2
Married	7	5.8
Stable union	6	4.9
Widowed	4	3.4
**Work situation**		
Unemployed	81	66.9
Informal job	32	26.4
Retired	5	4.2
Formal job	3	2.5
Monthly income*		
Up to 1 minimum wage	75	62.9
No income	29	23.1
1-3 minimum wages	17	14.0
**Home**		
Street situation	91	75.3
House/Apartment/Dormitory	23	19.0
Admission unit	6	4.9
Other	1	0.8

*Minimum wage to be considered: R$ 998.00 – Brazil, 2019

Regarding use of psychoactive substances, the users reported a mean of 25 years (SD=11.8), especially licit ones [alcohol (81-8%; n=99) and tobacco (83.6%; n=101)]. In times of crisis, they sought care at the CAPS AD III with demands related to a desire to reduce consumption (82.6%; n=100), support for detoxification (71.1%; n=86) and support for living in a situation of extreme social vulnerability, especially on the street (47.1%; n=57).

Fourteen days after comprehensive admission (T_1_), there was a significant reduction in the consumption of alcohol, marijuana and crack (<0.001) and an increase in days using tobacco and on withdrawal. As presented in Table2, consumption was evaluated based on the last 30 days.

**Table 2 -:** Days of psychoactive substance consumption before and after admission in a crisis at the Psychosocial Care Centers specialized in Alcohol and Other Drugs III. São Paulo, SP, Brazil, 2019

Consumption in the last 30 days	Mean days at admission T_0_ (n=121)	Mean days after admission T_1_ (n=67)	p-value
Days on withdrawal	8.81	14.75	<0.001*
Alcohol	20.80	12.72	<0.001*
Tobacco	22.38	25.30	0.079
Marijuana	8.83	4.39	<0.001*
Inhaled cocaine	7.18	6.24	0.203
Crack	7.76	4.13	<0.001*
Inhalants	1.13	0.12	0.070
Benzodiazepines	0.48	0.45	0.960

*p<0.001; Linear regression of mixed effects

In the follow-up period, we identified that most of the users [94% (n=63)] remained the 14 days foreseen in comprehensive admission at the CAPS AD III, as well as that they reported that this resource contributed positively to face the crisis moment. In addition to that, 79% (n=53) asserted that their requirements/needs were met.

As for the referrals described in Figure1, of the 67 users who were admitted in a crisis in the ten months of the research and who finished the follow-up period, nine (13.4%) required external support from the health network and were referred to other services. This result presented no significant difference in the follow-up period (p=0.470) (generalized linear model of mixed effects).

Consequently, the CAPS AD III’s ability to handle crises was 86.6%, evaluated as positive (less than 20% referrals), using the following calculation: 9 (number of users referred)/67 (number of users admitted)=0.134 or 13.4%.

**Figure 1 -:** Referrals of users admitted in crisis at the Psychosocial Care Centers specialized in Alcohol and other Drugs. São Paulo, SP, Brazil, 2019

**Month**	**Number of users admitted** **(T**_1_ **)**	**Referrals**	**Referral locus**	**Reason(s)**
**Feb.**	4	0	-	-
**Mar.**	9	1	General Hospital	Pneumonia
**Apr.**	11	2	Basic Health Unit Urgency Care	Anemia (pregnant woman) Pleural effusion
**May**	12	2	General Hospital Emergency Unit	Surgery HIV* infection
**Jun.**	5	2	Emergency Unit Emergency Unit	Suspected dengue Severe Alcohol Withdrawal Syndrome
**Jul.**	2	0	-	-
**Aug.**	6	0	-	-
**Sep.**	5	0	-	-
**Oct.**	6	1	Emergency Unit	Attempted suicide
**Nov.**	7	1	Psychiatric Hospital	Psychological disorganization associated with substance use
**Total**	67	9		

*HIV = Human Immunodeficiency Virus

The characteristics of the users referred to other health services are presented in Table3.

**Table 3 -:** Characteristics of the users admitted in crisis and referred to other health services. São Paulo, SP, Brazil, 2019

Variables	Mean	Standard Deviation
**Age (years old)**	42.3	8.1
**Schooling (years)**	6	1.8
**Consumption of substances (last 30 days)**	23	4.2
	**Frequency**	
	**n (9)**	**%**
**Gender**		
Male	6	66.7
Female	3	33.3
**Race/Skin color**		
Brown	6	66.7
Indigenous	1	11.1
Black	1	11.1
White	1	11.1
**Marital status**		
Single	7	77.8
Divorced	1	11.1
Married	1	11.1
**Work situation**		
Unemployed	6	66.7
Informal job	3	33.3
**Street situation**		
Yes	7	77.7
No	2	22.2
**Monthly income**		
Less than 1 minimum wage*	9	100
**Receiving social benefits**		
Yes	8	88.9
No	1	11.1

*Minimum wage to be considered: R$ 998.00 – Brazil, 2019

## Discussion

By evaluating the ability of the CAPS AD III in downtown São Paulo to handle crises arising from the problematic use of AOD, this study found promising results that reinforce the quality of psychosocial assistance in mental health care in emergencies, in territories of extreme social vulnerability.

Among the users admitted in different crises, 13.4% required external referral, which evidenced the ability of the services to handle these situations in their own territory. This result is corroborated by international studies, which evidenced that most of the crisis demands in mental health are solved by community services or specialized teams in the community^(4,20)^. As an example, a study in Switzerland identified 12.7% of referrals for hospitalization^(20)^. We did not find studies that evaluated the CAPS AD III’s ability to handle crises.

The profile of the participants included in the current study is similar to that of other studies on comprehensive care in CAPS AD III: men, homeless, with low schooling and problematic use of alcohol, tobacco, and crack^(2,7,10)^. This profile differs from other contexts since, in many countries from North America, the United Kingdom, and Europe, special attention is given to crisis management actions for opioid users and their consequences^(3,21)^.

Situations that resulted in referral by the CAPS AD III were largely associated with physical health problems. This result is expected for this type of care, as these services lack the structural and human resources to deal with emergencies of this nature. In addition to that, among the three referrals associated with deterioration in substance use or mental health problems, only one required psychiatric hospitalization.

Intoxication due to the use of AOD is one of the demands that most justifies clinical or psychiatric referral of users assisted in crisis in the community services, accounted for between 28.4% and 40% of the cases^(14,20)^. In addition to that, referrals often occur directly to hospitals without the patients receiving any other type of care^(21)^.

An analysis that reflects on the professionals’ conceptions identified that they sometimes do not recognize their responsibility for managing and welcoming a person in crisis at the CAPS III, believing that the role of this service as a central RAPS device is that only “stable” users be assisted. In contrast, people in times of psychological disorganization should be referred to health services such as general hospitals^(22)^.

On the other hand, consistent with the current study, an investigation into crisis care at the RAPS in Rio de Janeiro showed that several crises receive successful courses of action without the aid of closed psychiatric institutions, as the contact established in a network between the Urgency Medical Care Service (*Serviço de Atendimento Médico de Urgência*, SAMU), emergency care service, CAPS and Basic Health Unit (BHU) has been sufficient to deal with the management of critical situations in the territory and avoid long-term hospitalizations^(2)^.

The international scientific production highlights the need for a cultural change about what is meant by a crisis in mental health and how it should be addressed, as this is not just a crisis inherent to the person, but also to the context^(4,23)^. A number of research studies point out that the physical proximity of the services, the determinants of regular access to community care, and the preparation of the teams in this approach are essential to achieve positive results in crisis management in the community^(21-22)^.

In general, conceptions and ideals about crisis care are antagonistic, basing their mental health care protocols on different theoretical frameworks that can be organized into three categories: 1- Centralized care and a non-integrated network (for example, psychiatric hospitals); 2- Centralized care and integrated network (such as CAPS); and 3- Integrated and self-regulated network (performing crisis intervention in all health services). This conceptual opposition weakens the dissemination and expansion of deinstitutionalization, which further reinforces the importance of evaluating the model of territorial care with a psychosocial approach^(22)^.

A study that sought to identify actions of attention to the crisis and the meanings that involve them stated that, for structuring intervention policies during crises in mental health, three axes must be considered: territory, care and accountability^(24)^. In the context of CAPS AD, we include Harm Reduction (HR) as a fourth axis. This approach takes place in the territory through co-responsibility between professional and user, respects the rights and encourages the autonomy of the person who is in care, even if in times of crisis, not reducing the complexity of the situations that permeate the crisis to the withdrawal need and/or requirement^(25)^.

In the CAPS, there is space for immediate care for serious situations, although mainly for the needs that develop over the 14 days in comprehensive admission, as the crisis is not one-off since it also manifests itself in the detoxification process, generating intense and lasting anxiety, associated with abstinence or craving for substance use, as well as in discharge planning when the user’s return to the life context is conditioned by vulnerabilities, which deserves special attention from health teams^(22,24)^.

Users admitted at the CAPS AD III are exposed to stressful and conflicting factors. In their daily lives, they experience situations of humiliation, exclusion, precariousness, unmet basic needs, the impossibility of exercising citizenship, housing problems and fragile and/or non-existent family and social ties, in addition to countless risk situations that can trigger a mental health crisis, even when there is a need for protection, a possible alternative with comprehensive admission^(10)^.

Considering this complexity, a strategy adopted in Trieste (Italy) is to develop a program that seeks to monitor users who present signs or have already experienced crisis situations and were admitted by community services. The team discusses and updates a document daily, according to the perception of crisis risk. Detailed information is evaluated (different manifestations, substance use, major events, etc.) along with possible preventive or care continuity interventions. The main objective of this program is to offer daily and singular care, closer and more flexible, so that users are fully assisted in their territory and, if they happen to present crises, these are overcome with less distress intensity^(4)^.

Comprehensive admission is a fundamental tool to allow users to experience and go through the crisis moment with safety and freedom, without further harm. The gateway for this care is considered a strategic path in the network to accommodate the demand since, with 24-hour assistance, users can benefit from all the resources offered by the services^(1)^. In addition to that, the presence of Nursing teams, including weekends, and the support of peers in the community services, can contribute to the good result of management in crises, with a significant improvement in the biopsychosocial aspects^(8)^.

The break to take care of the crisis can be considered a clinical act. A strategy that opens up conditions for the exchange, speaking and listening to the subjects, and consequent resumption of possibilities. It represents an opportunity for users to take care of the distress caused by problematic AOD use and social exclusion^(1)^. In this sense, dialogue and establishing a relationship of trust offered in this device can ease approaches to users in crisis, allowing professionals and the person in care to access in more depth the relationship between consumption and care planning, in addition to medication support^(4)^.

From this perspective, many studies point to some benchmarking parameters for attention to crises, of which we highlight the following:

1. Interventions require a differentiated expenditure of time and energy when compared to conventional demands, requiring teams to be more flexible in devising responses; 2. Many users have autonomy about the need to circulate on the network. For these cases, the services must remain open as a reference to be accessed based on the subjects’ desire; and 3. Different negotiations must be allowed in each situation^(1,4)^.

The current study contributes to scientific progress by proposing to adapt an indicator to assess the CAPS AD III’s ability to handle crises. This result is relevant because it generates evidence that opposes questions about the efficacy of community mental health services based on managing crises. With this research, it is possible to promote reflections on the directions of public policies for the use of psychoactive substances and evaluations in this field.

As study limitations, we have a high research discontinuation rate among the users in the follow-up period (T_1_), partly expected for a follow-up study with this population segment, as well as the absence of a defined evaluative parameter for the indicator when assessing crisis management by CAPS III, which can generate different interpretations by researchers, and the need to adapt the indicator for CAPS AD III.

These services have different specificities, and in the context of substance use and the network support for the care of physical manifestations resulting from intoxication or detoxification should be considered more frequently; finally, it was not investigated how to care for crises was conducted by the services evaluated, as well as the variables that can be associated with the referrals, important objects for future research studies.

## Conclusion

It is concluded that the CAPS AD III evaluated in downtown São Paulo is capable of handling the users’ crises in their territory with the resource of comprehensive admission. These services maintain articulation with the network for forwarding demands beyond their specificity, thus achieving deinstitutionalization objectives. The profile and life context of the users included in the study should be considered, as the condition of social vulnerability, especially the homeless situation, seems to influence the result of this indicator directly.
